# Enhancing Electrical Outputs of Piezoelectric Nanogenerators by Controlling the Dielectric Constant of ZnO/PDMS Composite

**DOI:** 10.3390/mi12060630

**Published:** 2021-05-28

**Authors:** Yerkezhan Amangeldinova, Dimaral Aben, Xiaoting Ma, Heesang Ahn, Kyujung Kim, Dong-Myeong Shin, Yoon-Hwae Hwang

**Affiliations:** 1 Department of Nano Energy Engineering & BK 21 PLUS Nanoconvergence Technology Division, Pusan National University, Busan 46241, Korea; yamangeldinova@pusan.ac.kr (Y.A.); daben@pusan.ac.kr (D.A.); 2 Department of Mechanical Engineering, The University of Hong Kong, Pokfulam, Hong Kong 999077, China; xtma_2020@connect.hku.hk; 3 Department of Optics and Mechatronics Engineering, Pusan National University, Busan 46241, Korea; ahn3890@gmail.com (H.A.); k.kim@pusan.ac.kr (K.K.)

**Keywords:** nanogenerator, piezoelectricity, dielectric constant, ZnO

## Abstract

Structural optimizations of the piezoelectric layer in nanogenerators have been predicted to enhance the output performance in terms of the figure of merit. Here, we report the effect of dielectric constant on electrical outputs of piezoelectric nanogenerator using ZnO/PDMS composites with varied ZnO coverages. The dielectric constant of piezoelectric layers was adjusted from 3.37 to 6.75. The electrical output voltage of 9 mV was achieved in the nanogenerator containing the ZnO/PDMS composite with the dielectric constant of 3.46, which is an 11.3-fold enhancement compared to the value of the nanogenerator featuring the composite with high dielectric constants. Significantly, lowering the dielectric constant of the piezoelectric layer improves the electrical output performance of piezoelectric nanogenerators.

## 1. Introduction

Ever since the conceptual study of piezoelectric nanogenerators (PENGs) in 2006 [1], most researchers have focused on nanogenerators featuring ZnO nanostructures due in a large part to their non-toxicity, biocompatibility, and sensitivity to tiny strains. In their early development, the performance of nanogenerator was governed by the Schottky contact at the metal–ZnO interface. However, the Schottky barrier concept is problematic especially for sustainability, reproducibility, processability, and uniformity, and has slowed down improvements in the performance of such devices. A milestone in 2010 [2], which was the integration of dielectric barriers into devices, shifted the paradigm for the structural design of devices. Subsequently, the effective integration of ZnO nanorod arrays dramatically increased the output performance up to 4.4 W m^−2^ [3]. Successes in nanogenerators comprising ZnO nanostructures have driven the development of many advanced piezoelectric materials [4,5,6,7,8,9,10,11]. Piezoelectric materials, which have been adopted in nanogenerators, can be categorized into two different groups: inorganic and organic materials. Specifically, two-dimensional inorganic and organic materials have been of great interest, as they fulfill the needs of flexible energy harvesting. Some promising piezoelectricity includes MoS_2_ [12] and WSe_2_ [13], with piezoelectric coefficients of ~3 pm V^−1^ and 3.4 pm V^−1^, respectively. After stacking many units using an efficient approach was found to greatly enhance the output, PbI_2_ [14] and hexagonal-BN [15] were used as active materials for nanogenerators. Researchers have also demonstrated piezoelectric polymers [16] and biomaterials [17,18,19] with piezoelectric coefficients in the range of ~7.8 to 30 pm V^−1^.

Apart from the material studies, structural optimizations of PENGs have been proposed to improve the performance in terms of the figure of merit (FOM) using the relationship of the open-circuit voltage, *V_oc_*, of the piezoelectric layer as follows:

$$V_{OC}=\frac{d_{ij}}{\varepsilon_{r}\varepsilon_{0}}\sigma_{ij}g_{e}$$

where *d_ij_* is the piezoelectric coefficient, *σ_ij_* is the applied stress, *g_e_* is the gap distance between electrodes, *ε_r_* is the relative dielectric constant, and *ε*_0_ is the vacuum permittivity [20]. Control over the relative dielectric constant of the piezoelectric layer is predicted to be a factor in enhancing the FOM, and ultimately, the high open-circuit voltage. Herein, we investigated the effect of dielectric constant modulation on the electrical output of piezoelectric nanogenerators featuring a ZnO/polydimethylsiloxane (PDMS) composite. The porous ZnO membranes were fabricated by calcinating the ZnO/poly(methyl methacrylate) (PMMA) composite, and the elastomeric PDMS was infiltrated into the porous ZnO membrane to form the ZnO/PDMS composite. Our approach to control the dielectric constant of the ZnO/PDMS composite relied on regulating the porosity of the ZnO membrane. The dielectric constant of the piezoelectric composite was successfully modulated from 3.37 to 6.75 by changing the ZnO concentration in the ZnO/PMMA composite. The output voltage of the piezoelectric nanogenerator was shown to improve approximately 11.3-fold by lowering the dielectric constant of the composite, which is well matched with our simulation results.

## 2. Experimental Section

### 2.1. Fabrication of ZnO/PDMS Composite and Nanogenerators

**Figure 1:**
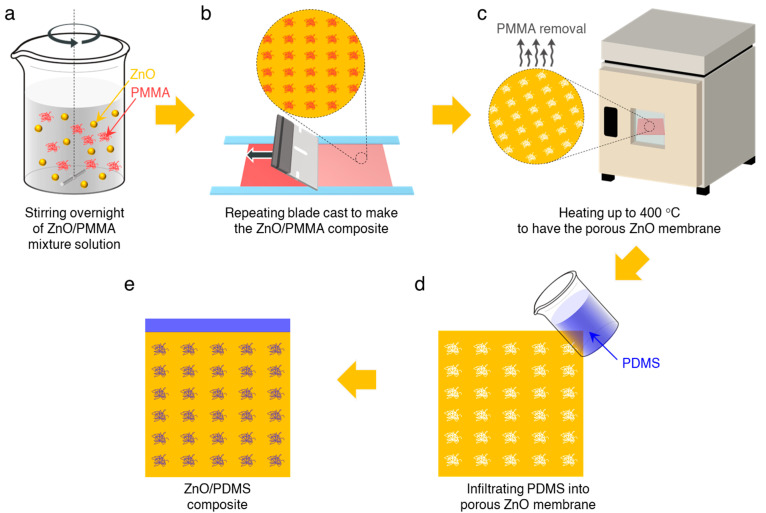
Schematic illustration of the ZnO/PDMS composite fabrication. (**a**) Preparation of ZnO/PMMA precursor. (**b**) Blade casting of ZnO/PMMA composite. (**c**) PMMA removal by thermal annealing. (**d**) Infiltration of PDMS into ZnO membrane. (**e**) ZnO/PDMS composite.

A schematic illustration of a homogenous ZnO/polydimethylsiloxane (PDMS) composite is displayed in Figure 1. The ZnO powder was purchased from Sigma Aldrich and was used without further purification. The precursor solutions composed of ZnO powder in ethanol (15% *w*/*w*) and poly(methyl methacrylate) (PMMA, Sigma Alrich) in toluene (20% *w*/*w*) were stirred for 24 h (Figure 1a), and then were blade-casted onto the gold-coated glass substrate (Figure 1b). The ZnO/PMMA composites were heated to 75 °C for 10 min to evaporate the volatile solvent. The blade casting and subsequent drying process were repeated thrice. The ZnO/PMMA composites were heated to 400 °C for 4 h in a furnace to yield the porous ZnO membranes by calcinating PMMA (Figure 1c). Next, the porous ZnO membranes were immersed in a PDMS (SYLGARD™ 184, Dow Chemical) elastomeric layer (Figure 1d), which served not only as a potential barrier, but also as a buffer layer to improve the robustness and durability of the ZnO/PDMS composite, followed by curing at 70 °C for 120 min (Figure 1e). We deposited an adhesion layer of 5-nanometer-thick chromium (Cr), followed by a 100-nanometer-thick gold (Au) film using a radio frequency sputter. The Au films positioned at the top and bottom surfaces of the ZnO/PDMS composite serve as the electrodes for piezoelectric nanogenerators. Then, the composite with electrodes was immersed in a polydimethylsiloxane (PDMS) elastomeric layer.

### 2.2. Characterization

The morphologies of the composite were observed using a field-emission scanning electron microscope equipped with energy dispersive X-ray spectrometer (Hitachi S-4700), and X-ray diffraction (Xpert, 2016) analysis was performed to check the crystallinity of the ZnO after annealing. Adobe photoshop image analysis ‘count tool’ was used to determine the porosity of ZnO templates and study the ZnO and PDMS coverage of the composites. The dielectric constant was characterized using a broadband dielectric spectrometer (Concept 80, Novocontrol Corp). The output performance of PENGs was measured using an oscilloscope (Agilent DSO-X-2014A) equipped with a current preamplifier (SRS SR- 570) under different pushing loads applied by a customized pushing tester driven by a linear motor (LS Mechapion APM-SB02ADK). The output voltage was measured with a direct connection to a standard probe with 10 MΩ resistance, while the output current was recorded with a probe with 50 Ω resistance. The power of the system was calculated using varied load resistance. The COMSOL Multiphysics was used to simulate the piezoelectric potential in the ZnO/PDMS composite with varying ZnO coverage.

## 3. Results and Discussion

**Figure 2:**
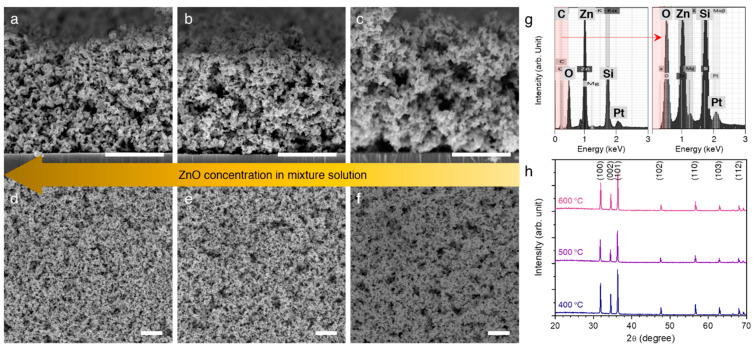
Porous ZnO membrane. (**a**–**c**): Cross-sectional SEM images of porous ZnO membranes made from the precursor solutions of varying ZnO concentrations from 0.3 to 1 M. Scale bar = 5 μm. (**d**–**f**): Surface morphology of the porous ZnO membranes. Scale bar = 5 μm. (**g**): EDX spectrum of the porous ZnO membrane before (left) and after (right) the calcination. (**h**): XRD results of the porous ZnO membranes annealed at different temperatures.

The scanning electron microscopy (SEM) images presented in Figure 2a–f show the typical morphologies of the porous ZnO membranes made from the precursor solutions of varying ZnO concentrations from 0.3 to 1 M. As the ZnO concentrations decreased, more PMMA agglomerates were trapped in the ZnO membranes, and then the sacrificial PMMA agglomerates were completely removed after calcination at 400 °C for 4 h. It is worth noting that the ZnO particles are known to be homogeneously dispersed in the PMMA matrix [21,22,23], facilitating the formation of the homogenous porous ZnO membrane. The SEM images indicate that the porous ZnO membranes, with thicknesses ranging from 8.7 to 11.3 μm, were successfully achieved over a large scale as high as 65 × 25 mm^2^, and the average volumetric density of ZnO in a given region considerably increased as a result of controlling the ZnO concentrations. Using energy-dispersive X-ray spectroscopy (Figure 2g), we confirmed that the carbon concentrations in the porous ZnO membrane decreased significantly after the calcination, indicating that the sacrificial PMMA was successfully removed from the ZnO/PMMA composite. The crystalline structures of the porous ZnO membrane of differing annealing temperatures from 400 to 600 °C were investigated using X-ray diffraction (XRD), as shown in Figure 2h. All of the diffraction peaks corresponding to (100), (002), (101), (102), (110), (103), and (112) planes belong to the standard pattern for ZnO powder, and can be indexed to the wurtzite structure of ZnO with lattice constants of a = 3.248 and c = 5.199 Å, which is in accordance with the reported values (a = 3.25 and c = 5.207 Å) in the JCPDS files (card no. 36-1451). The intensity ratio of (100)/(002) and (101)/(002) planes was calculated and is given in Table 1. A low annealing temperature resulted in the highest relative ratios of 1.50 and 2.15, respectively, with an increase in annealing temperature decreasing the relative ratios down to 1.09 and 1.17, respectively. We then calculated the lattice aspect ratio using the full width at half maximum of the (100) and (002) diffraction peak in Scherrer’s equation. The results indicated that the lattice aspect ratio was negligibly improved with increasing the annealing temperature, so that the subsequent measurements described below using the porous ZnO membranes were all performed with the samples annealed at 400 °C.

**Figure 3:**
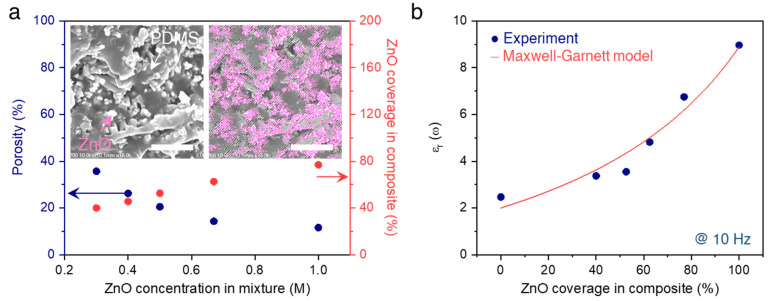
ZnO coverage and dielectric constant of the ZnO/PDMS composites. (**a**): porosity and ZnO coverage in the composite as a function of the ZnO concentration in the precursor solution. (**b**): Dielectric constant of the ZnO/PDMS composites. The data of 100% ZnO coverage and 0% ZnO coverage (fully covered by PDMS) were extracted from the literature: PDMS [25] and ZnO [26].

We employed the image analysis technique to further investigate the approximate porosity of ZnO membranes in the projected SEM images (Figure 3a, left). The white and dark regions were considered as the ZnO membrane and voids, respectively, and then the area of each region was calculated by counting the number of pixels. Remarkably, the porosity, representing the fraction of ZnO in the given area, was enhanced up to 35.7% as the ZnO concentration in the precursor solutions was decreasing. After infiltrating the PDMS into the porous ZnO membranes, the approximate ZnO coverages, Coverage_ZnO_ = A_ZnO_/(A_ZnO_ + A_PDMS_), where A_ZnO_ and A _PDMS_ denote the area of ZnO and PDMS, respectively, in the ZnO/PDMS composites were also computed in a similar manner (Figure 3a, right). The white and grey regions were taken into account as the ZnO membrane and PDMS infiltrated, respectively (Inset of Figure 3a). The ZnO coverage is shown to increase with the decreasing of the porosity, and the ZnO coverage shrunk down to 40% in the ZnO membrane with a porosity of 35.7%, representing the successful infiltration of PDMS into the porous ZnO membrane. A broadband dielectric spectrometer was used to characterize the dielectric constant of ZnO/PDMS composites in a symmetric Au | ZnO/PDMS composite | Au configuration, as shown in Figure 3b. The experimental dielectric constant substantially increased with the ZnO coverage at 10 Hz. We estimated the dielectric constant of the composites using theoretical models developed by Maxwell Garnett, which is suitable for a two-phase composite and given by [24]:

$$\varepsilon_{composite}=\varepsilon_{PDMS}\frac{2\varepsilon_{PDMS}+\varepsilon_{ZnO}+2f_{ZnO}\left(\varepsilon_{ZnO}-\varepsilon_{PDMS}\right)}{2\varepsilon_{PDMS}+\varepsilon_{ZnO}-f_{ZnO}\left(\varepsilon_{ZnO}-\varepsilon_{PDMS}\right)}$$

where *f_ZnO_* is the volume content of ZnO in the composite, and *ε_PDMS_*, *ε_ZnO_*, and *ε_composite_* represent the dielectric constant of the PDMS region, ZnO region, and composites, respectively. The relative permittivity of PDMS (*ε_PDMS_*) and ZnO (*ε_ZnO_*) are known as 2.47 and 8.96, respectively, according to the previous studies [25,26]. It can be noticed that the Maxwell Garnett model yields a similar prediction of *ε_ZnO_* around 8.89, with a coefficient of determination, R^2^, very close to 1, reflecting an excellent correlation between the empirical data and estimated values. All the above results allow us to confirm that the dielectric constant of composites was clearly modified from 3.37 to 6.75 by modulating the ZnO coverage in the ZnO/PDMS composite.

**Figure 4:**
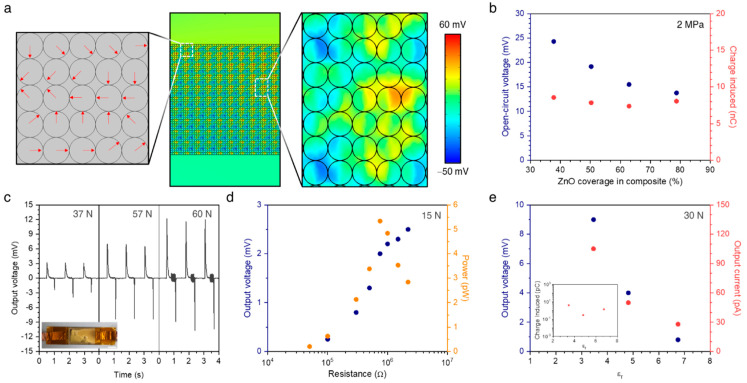
Effect of dielectric constant on piezoelectric output. (**a**): computed potential distribution in ZnO/PDMS composite with a 78.7% ZnO coverage under a compressive pressure of 2 MPa. (**b**): computed open-circuit voltage and charge simulated of ZnO/PDMS composites. (**c**): Output voltage of nanogenerator under different compressive loads. Inset indicates the photo of nanogenerator fabricated. Scale bar = 1 cm. (**d**)**:** output voltage and power from nanogenerator as a function of different resistors as external loads. (**e**): Output voltage and output current as a function of dielectric constant. Inset indicates the charge values extracted from the integration of a single current peak.

In order to have an insight into the distribution of the electric field within the ZnO/PDMS composite, a two-dimensional finite element method simulation was carried out. The ZnO circular particles with a diameter of 200 nm were embedded in the PDMS matrix with a 20 μm width and 10 μm thickness. The ZnO coverage was controlled by varying the distance between particles while maintaining the particle size. Therefore, the number of ZnO particles for 78.7% ZnO coverage was 6250, and this value decreased to 5000, 4000, and 3000 in the ZnO coverages of 63%, 50.4%, and 37.8%, respectively. To represent the crystalline direction of the ZnO nanoparticles, we set the unit cell consisting of the ZnO/PDMS composite with a dimension of 1 μm × 1 μm, where the dominant polarization directions of each particle were randomly assigned (Figure 4a, left). Then, the imaginary circle, which was much larger than our sample, was set to be grounded as the zero potential references. The floating potential with zero initial potential was designated for both the top and bottom surfaces of the ZnO/PDMS composite. Upon the compressive force to the top surface, the ZnO/PDMS composite can be freely deformed in both lateral and vertical directions, while the movement of the bottom surface was restricted in a vertical direction. Figure 4a illustrates the electric field distribution in the ZnO/PDMS composite with a 78.7% ZnO coverage under a compressive pressure of 2 MPa. The ZnO/PDMS composite showed the random potential distribution, which is attributed to the random orientation of polarization induced by ZnO particles, but the net polarization induced the potential difference between the top and bottom electrodes. The open-circuit voltage was calculated from the potential difference between the top and bottom surfaces of the composite, and is presented in Figure 4b. Notably, the open-circuit voltage was found to increase gradually with decreasing ZnO coverage (i.e., lowering dielectric permittivity), whereas the charge induced by the piezo-potential plateaus for all ZnO coverages, implying a decrease in dielectric constants, enabled them to improve the output voltage of the nanogenerator in terms of the figure of merit.

We constructed the piezoelectric nanogenerators comprising the ZnO/PDMS composites with different ZnO coverages. The ZnO/PDMS composites acted as a piezoelectric material, and the top and bottom electrodes were deposited on both surfaces of the composite using a thermal evaporator. Encapsulation with an additional PDMS layer provides high structural stability of the nanogenerators. The dimensions of our device were 65 × 25 mm^2^ in the area and ~100 μm in thickness (Inset of Figure 4c). Each nanogenerator included the ZnO/PDMS composite with an almost similar thickness of 23–34 μm. The output voltage and current were measured by applying periodic compressive loads using a programmed linear motor. Under a compressive stimulus (37 N) perpendicular to the nanogenerator, the output voltage of the nanogenerator featuring composites with 52.6% ZnO coverage reached up to ~3.2 mV (Figure 4c). The electrical output increased linearly with applied loads from 30 N to 60 N. Then, the maximum peak power of the nanogenerator was investigated using different resistors, with a value of 5.34 pW at a load resistance of approximately 0.75 MΩ in the presence of 15 N external load (Figure 4d). We then investigated the effect of the dielectric constant of composites on the electrical output (Figure 4e). The electrical output voltage of 0.8 mV was achieved in the nanogenerator involving the ZnO/PDMS composite with the dielectric constant of 6.75, and this value increased to 4 and 9 mV in those with the dielectric constants of 4.81 and 3.46, respectively. The nanogenerator featuring the ZnO/PDMS composite with the highest dielectric constant produced an average output current of 28 pA, and the current was shown to increase up to 105 pA with the lowering of the dielectric constant. The charge values were extracted from the integration of a single current peak and the charge values plateau occurred for all dielectric constants, which exhibited good agreement with our simulation results. Ultimately, control over the relative dielectric constant of the piezoelectric layer is a promising approach for enhancing the FOM, and ultimately, the high open-circuit voltage.

## 4. Conclusions

The effect of the dielectric constant of the piezoelectric layer on electrical outputs of the piezoelectric nanogenerator was investigated using ZnO/PDMS composites with differing ZnO coverage. We adjusted the porosity of the ZnO membrane from 11.6% to 35.7% by changing the ZnO concentration in the precursor solution, and then the elastomeric PDMS was permeated into the porous ZnO membrane, yielding the ZnO/PDMS composites with the dielectric constants in the range of 3.37 and 6.75. The output voltage of the piezoelectric nanogenerator comprising the piezoelectric composite with the lowest dielectric constant was found to be boosted approximately 11 times compared to that of the nanogenerator with the highest dielectric constant. Additionally, the results found in experiments were well matched with those of the simulation study.

## Figures and Tables

**Table 1:** Geometric parameters of the ZnO nanostructures calculated from XRD spectra.

Annealing Temperature (°C)	Crystalline Size (nm)	Relative Intensity		XRD Lattice Aspect Ratio
		(100)/(002)	(101)/(002)	
400	58.9	1.50	2.15	1.00
500	53.9	1.15	1.29	1.01
600	48.5	1.09	1.17	0.99
